# The acute effect of exercise intensity on peripheral and cerebral vascular function in healthy adults

**DOI:** 10.1152/japplphysiol.00772.2021

**Published:** 2022-07-07

**Authors:** Max E. Weston, Jodie L. Koep, Alice B. Lester, Alan R. Barker, Bert Bond

**Affiliations:** ^1^Children’s Health and Exercise Research Centre, Sport and Health Sciences, College of Life and Environmental Sciences, University of Exeter, Exeter, United Kingdom; ^2^Physiology and Ultrasound Laboratory in Science and Exercise, School of Human Movement and Nutrition Sciences, University of Queensland, Brisbane, Queensland, Australia

**Keywords:** cerebrovascular reactivity, endothelial function, flow-mediated dilation, HIIE

## Abstract

The acute effect of exercise intensity on cerebrovascular reactivity and whether this mirrors changes in peripheral vascular function have not been investigated. The aim of this study was to explore the acute effect of exercise intensity on cerebrovascular reactivity (CVR) and peripheral vascular function in healthy young adults (*n* = 10, 6 females, 22.7 ± 3.5 yr). Participants completed four experimental conditions on separate days: high-intensity interval exercise (HIIE) with intervals performed at 75% maximal oxygen uptake (V̇o_2max_; HIIE1), HIIE with intervals performed at 90% V̇o_2max_ (HIIE2), continuous moderate-intensity exercise (MIE) at 60% V̇o_2max_ and a sedentary control condition (CON). All exercise conditions were completed on a cycle ergometer and matched for time (30 min) and average intensity (60% V̇o_2max_). Brachial artery flow-mediated dilation (FMD) and CVR of the middle cerebral artery were measured before exercise, and 1- and 3-h after exercise. CVR was assessed using transcranial Doppler ultrasonography to both hypercapnia (6% carbon dioxide breathing) and hypocapnia (hyperventilation). FMD was significantly elevated above baseline 1 and 3 h following both HIIE conditions (*P* < 0.05), but FMD was unchanged following the MIE and CON trials (*P* > 0.33). CVR to both hypercapnia and hypocapnia, and when expressed across the end-tidal CO_2_ range, was unchanged in all conditions, at all time points (all *P* > 0.14). In conclusion, these novel findings show that the acute increases in peripheral vascular function following HIIE, compared with MIE, were not mirrored by changes in cerebrovascular reactivity, which was unaltered following all exercise conditions in healthy young adults.

**NEW & NOTEWORTHY** This is the first study to identify that acute improvements in peripheral vascular function following high-intensity interval exercise are not mirrored by improvements in cerebrovascular reactivity in healthy young adults. High-intensity interval exercise completed at both 75% and 90% V̇o_2max_ increased brachial artery flow-mediated dilation 1 and 3 h following exercise, compared with continuous moderate-intensity exercise and a sedentary control condition. By contrast, cerebrovascular reactivity was unchanged following all four conditions.

## Graphical abstract

**Figure:**
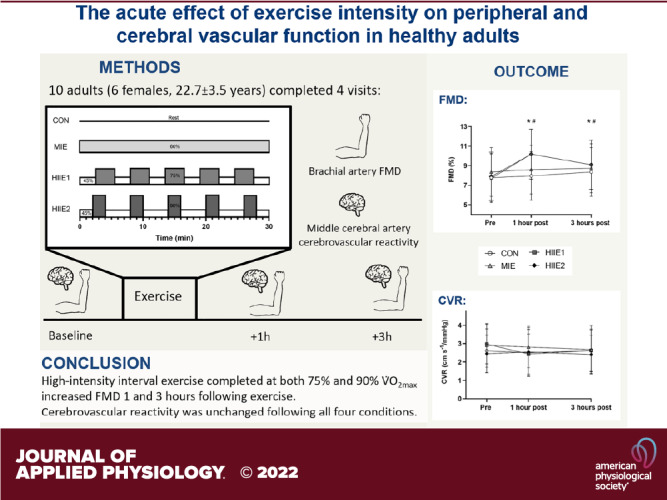

## INTRODUCTION

Atherosclerosis is a precursor to overt cardiovascular disease, and endothelial dysfunction is the first detectable manifestation of the atherosclerotic process (1). Aerobic exercise training is known to have beneficial effects on endothelial function (2), and this is mediated by exercise-induced increases in shear stress (3). Since the chronic benefits of exercise are likely related to the repeated acute responses following a single bout of exercise (4), it is important to investigate changes in endothelial function following a single bout of exercise (5). Increases in peripheral shear stress are greater with higher-intensity exercise (6, 7) and might therefore confer acute intensity-dependent improvements in peripheral vascular function (8, 9). However, the acute effect of exercise intensity on cerebrovascular reactivity has received little investigation.

Cerebrovascular function plays an important role in the risk of cerebrovascular diseases such as stroke, dementia, and cognitive decline (10, 11). Recently, Bliss et al., (12) highlighted the beneficial effects of exercise training on cerebrovascular health, including improved endothelial function and cerebral angiogenesis. High-intensity interval training is known to improve peripheral vascular function (13), and there is a growing interest in the effects of high-intensity interval exercise (HIIE) on cerebrovascular health (14–16). A commonly utilized measurement of cerebrovascular function is the ability of the cerebrovasculature to vasodilate or constrict in response to hypercapnia and hypocapnia, respectively, termed cerebrovascular reactivity (CVR). However, the acute effects of exercise on CVR are not well understood. In particular, it is not known whether acute changes in peripheral endothelial function (assessed through brachial artery flow-mediated dilation; FMD) are mirrored by changes in CVR. Initial evidence suggested the two may be related, since the overnight changes in FMD and CVR were strongly, positively correlated (17). However, more recently, no relationship has been observed between resting cerebral and peripheral vascular function in healthy young adults, suggesting the two may share different mechanistic pathways (18). Exploring whether peripheral vascular function and CVR respond similarly following an acute challenge (such as exercise) will provide further insight into whether they share a common mechanism of change (19). Currently, it is unknown if the acute effects of exercise on peripheral vascular function are mirrored by changes in CVR.

A recent systematic review explored the effect of HIIE on cerebrovascular function (16). In total, only seven eligible studies were found, which included a combination of acute and exercise training studies, with data on CVR following acute HIIE limited to a single study (20). In healthy adults, one bout of HIIE (completed at 85%–90% heart rate reserve) significantly lowered CVR to hypercapnia immediately and 1 h following HIIE but was restored to baseline levels 2 h following exercise (20). In contrast, moderate-intensity exercise and a sedentary control condition did not alter CVR. This was thought to be a result of repeated exposure to hyperventilation-induced hypocapnia during HIIE (21), impairing the dilatory capacity of the cerebrovasculature following exercise, which may explain why CVR did not fall following moderate-intensity exercise. However, the exercise conditions were not equivalent for time or work performed, which is an important consideration to isolate and understand the effects of exercise intensity (22).

Since shear stress appears to be the primary mechanism underlying acute (23) and chronic (3) exercise-induced improvements in peripheral vascular function, exercise that elicits the greatest increases in cerebral blood flow (CBF) may therefore result in the greatest postexercise improvements in CVR. During incremental cycling exercise, CBF increases until ∼75%–90% of maximal oxygen uptake (V̇o_2max_) (24) but then decreases with maximal-intensity exercise due to the role of hyperventilation-induced hypocapnia (25, 26). Prescribing HIIE at ∼75% V̇o_2max_ may therefore result in greater acute increases in CVR than exercise performed at a greater intensity, but this has not been explored.

This study aimed to investigate the acute effect of exercise intensity on peripheral and cerebral vascular function in healthy adults. Specifically, this study compared continuous moderate-intensity exercise (MIE, 60% V̇o_2max_), HIIE performed at 75% V̇o_2max_, and HIIE performed at 90% V̇o_2max_, which were all matched for time (30 min) and average intensity (target: 60% V̇o_2max_). It was hypothesized that *1*) both HIIE protocols would increase brachial FMD compared with MIE and a resting control; *2*) increases in middle cerebral artery blood velocity (MCAv) during exercise would be higher during HIIE completed at 75% V̇o_2max_ compared with MIE and HIIE completed at 90% V̇o_2max_; and *3*) CVR would be unchanged following MIE, increased following HIIE completed at 75% V̇o_2max_, and decreased following HIIE completed at 90% V̇o_2max_.

## METHODS

### Participants

An a priori sample size calculation was performed for this investigation. This study was powered to the intensity-dependent postexercise (1 h) changes in FMD, to detect an effect size of ∼1.2 (27). This revealed a required sample size of 12 participants.

Following ethical approval from the University of Exeter ethics committee (180613/A/07), 12 healthy adults volunteered to take part in this study. Exclusion criteria included smoking, contraindications to exercise, cardiometabolic disease, and the use of any medication or supplement known to influence vascular function. One participant did not complete the study due to an unrelated injury, and one participant was removed due to inadequate acquisition of vascular data. Consequently, data are presented as *n* = 10 (6 females) throughout. Participant characteristics are described in Table 1.

**Table 1.** Participant characteristics

Parameter	*n* = 10	Males (*n* = 4)	Females (*n* = 6)
Age, yr	22.7 ± 3.5	24.8 ± 5.1	21.3 ± 0.8
Body mass, kg	67.4 ± 9.7	73.8 ± 6.5	63.1 ± 9.4
Stature, m	1.70 ± 0.08	1.77 ± 0.08	1.66 ± 0.06
BMI, kg/m^2^	23.3 ± 2.1	23.7 ± 1.0	23.0 ± 2.7
V̇o_2max_, L/min	2.65 ± 0.50	3.18 ± 0.28	2.30 ± 0.19
V̇o_2max_, mL/kg/min	39 ± 5	43 ± 5	37 ± 4
V̇o_2max_ range, mL/kg/min	30–46		
Peak power, W	267 ± 40	309 ± 27	239 ± 13

Data are presented as the means ± standard deviation. BMI, body mass index; V̇o_2max_, maximal oxygen consumption.

### Study Design

Participants completed one preliminary visit and four subsequent experimental visits to the laboratory. The preliminary visit served to familiarize participants with all experimental procedures before participants completed an incremental (30 W/min) ramp test to exhaustion on an electronically braked cycle-ergometer (Lode Excalibur Sport, Groningen, the Netherlands). V̇o_2max_ was determined as the highest 10 s average (MedGraphics, UK). The mean V̇o_2_ response time from the incremental ramp test was accounted for when prescribing the power outputs for each exercise trial (28).

The four subsequent experimental visits were completed in a different order for each participant to control for any potential order effect. At least 48 h separated experimental visits, and the means ± SD time to complete the four visits was 25 ± 7 days. Participants were instructed to avoid vigorous exercise and alcohol consumption in the 24 h preceding each visit. Following an overnight fast (including abstaining from caffeine), participants reported to the laboratory at 0800 and were provided with a standardized cereal breakfast consisting of 50 g cornflakes and 150-mL semiskimmed milk. The macronutrient content is unlikely to have influenced vascular function (29, 30). Peripheral and cerebral vascular function were assessed 30 min after breakfast, and then 1 and 3 h after the completion of the experimental condition. Apart from the exercise trials, participants remained at rest in the laboratory throughout.

### Experimental Trials

**Figure 1.**
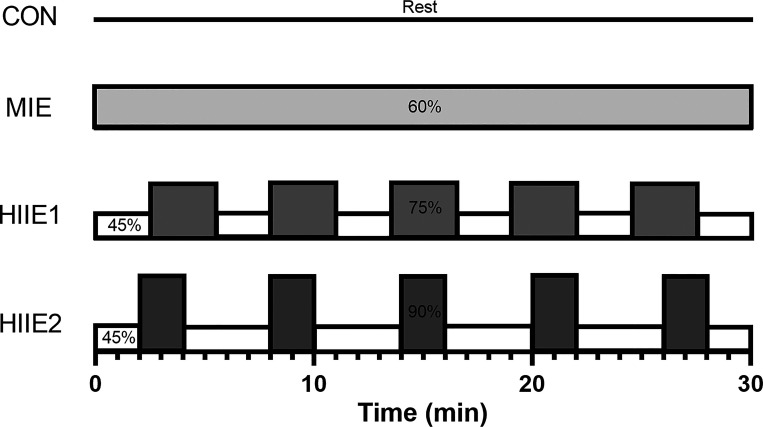
Experimental conditions. The numbers refer to cycling intervals at a percentage of maximal oxygen uptake for each individual. The average intensity of each exercise trial was designed to be 60% maximal oxygen uptake, and all exercise trials were 30 min in duration. CON, resting control trial; HIIE, high-intensity interval exercise; MIE, moderate-intensity exercise.

Immediately following the assessment of baseline resting vascular measures, participants completed either seated rested in the laboratory (control trial; CON), or 30 min of cycling at 60% V̇o_2max_ (moderate-intensity exercise; MIE), or two different 30 min HIIE protocols, with the work rate of the active intervals corresponding to either 75% (HIIE1) or 90% (HIIE2) V̇o_2max_. Specifically, HIIE1 included a 2.5-min warm-up and 2.5-min cooldown at 45% V̇o_2max_ and five 3-min intervals at 75% V̇o_2max_, interspersed with four 2.5-min recovery intervals at 45% V̇o_2max_. HIIE2 included the same 2-min warm-up and cooldown, and five 2-min intervals at 90% V̇o_2max_, separated by four 4-min recovery intervals at 45% V̇o_2max_ (Fig. 1). Each exercise protocol was completed using the same cycle-ergometer as the prior ramp test. The exercise conditions were matched for time (all trials were 30 min in duration) and average intensity (all trials were designed to have a target average intensity of 60% V̇o_2max_).

Breath-by-breath V̇o_2_ and end-tidal carbon dioxide ($\text{P}\text{E}{\text{T}}_{\text{C}{\text{O}}_{2}}$) during exercise were measured throughout (MedGraphics, UK) and averaged into 10-s time bins. Given the interval nature of HIIE1 and HIIE2, $\text{P}\text{E}{\text{T}}_{CO_{2}}$ data were also expressed as the highest (peak) and lowest (minimum) 10-s average during exercise. The velocity of blood in the middle cerebral artery (MCAv) was quantified via transcranial Doppler (TCD) sonography (MultiDop, DWL, Germany) using a 2 MHz probe placed over the temporal window and held in place using a customizable headset (DiaMon, DWL, Germany). MCAv data were sampled at a frequency of 200 Hz (PowerLab 8/30 ML880, ADInstruments) and then exported into 10-s time bins (LabChart version 8, ADInstruments), and 95% confidence intervals were calculated. The total area under the mean MCAv versus time (30 min) curve was calculated for each trial using the trapezoidal rule.

### Peripheral Vascular Function

Peripheral vascular function was quantified via FMD using high-resolution duplex ultrasonography (Apogee 1000, SIUI, China) with a 13 MHz linear array transducer, in accordance with current guidelines (31). Briefly, participants rested in a darkened, temperature controlled (∼23°C) room for 10 min before each assessment. Baseline diameter was determined over a 1-min period, which immediately preceded rapid (<0.3 s) forearm cuff inflation (Hokanson, Bellevue) to 220 mmHg for 5 min. The brachial artery was continuously imaged for 3 min after rapid deflation, and endothelial dependent (31, 32) vasodilation was calculated using the peak increase in arterial diameter. All images were assessed during end diastole using validated software (Brachial Analyzer for Research, MIA) (33), and analysis was performed blinded to the experimental condition. The area under the curve for shear rate was calculated from the point of cuff deflation until the time of peak dilation (SR_AUC_) (34). FMD was not normalized to SR_AUC_ as these were not consistently related, which is in line with other observations after exercise (35). The FMD statistic was allometrically scaled to address the observed changes in baseline diameter after exercise and the concerns regarding ratio scaling of this outcome (36). The within (pre- and post-CON) and between day (baseline of all four visits) coefficient of variation for the FMD statistic was 5.2% and 13.8%, respectively.

### Cerebrovascular Reactivity

Participants remained supine after the FMD protocol for ∼5 min before the assessment of cerebrovascular reactivity. MCAv was measured throughout using TCD (DWL, Germany) and end-tidal CO_2_ ($PET_{\text{C}{\text{O}}_{2}}$) using a gas analyzer (ADInstruments ML206). The depth and position of the probe were noted for each participant to standardize the insonation of the MCA for each participant within and between day (37). The within- and between-day coefficients of variation for baseline MCAv were 5.3% (95% CI: 3.8%–8.8%) and 9.3% (95% CI: 7.4%–13.2%), respectively. Following a 1-min recorded baseline, participants breathed 6% CO_2_, 21% O_2_, and balance nitrogen for 4 min. After 5 min of re-acclimatization, participants were then instructed to perform deep hyperventilation at a frequency of 25 breaths/minute for 1 min (CVR Hypocapnia) (38). To address recent concerns regarding the variability of changes in MCAv during open-circuit hypercapnic challenges (37, 39), CVR was quantified as the highest rolling 30-s average absolute change in MCAv per 1 mmHg increase in $\text{P}\text{E}{\text{T}}_{\text{C}{\text{O}}_{2}}$ (37). CVR to hypocapnia was quantified as the absolute change in MCAv from rest per 1 mmHg change in $\text{P}\text{E}{\text{T}}_{\text{C}{\text{O}}_{2}}$ in the final 10 s of hyperventilation. To account for the influence of potential changes in mean arterial pressure on CVR outcomes within and between day, beat-by-beat blood pressure was noninvasively measured via finger plethysmography (Human NIBP Nano, ADInstruments) during assessments of CVR. The ratio between resting mean arterial pressure and MCAv was expressed as the cerebrovascular conductance index (CVC = MCAv/mean arterial pressure). All MCAv, mean arterial pressure, and $\text{P}\text{E}{\text{T}}_{\text{C}{\text{O}}_{2}}$ data were integrated (Powerlab; model 8/30, ADInstruments) and stored at 200 Hz using an analogue-to-digital converter interfaced with a laptop computer (Lab Chart v. 8, ADInstruments).

### Statistical Analyses

Data are presented as means ± standard deviation (SD). All analyses were performed using SPSS version 26 (IBM). Differences in the physiological responses during each of the experimental trials were explored using a one-way ANOVA. All vascular responses were analyzed using a mixed model ANOVA, with trial (CON, MIE, HIIE1, HIIE2) and time (pre, 1 h post, 3 h post) as the main effects. Statistical significance was accepted when *P* < 0.05, and effect sizes were calculated to demonstrate the magnitude of any difference. Effect sizes for the ANOVA main and interaction effect were interpreted using partial eta squared ($\eta_{\text{p}}^{2}$) values of ≤0.06, small; 0.06–0.14, moderate; and >0.14, large (40). Follow-up pairwise comparisons were interpreted using standardized effect sizes (*d*): small <0.5, moderate 0.5–0.8, and large ≥0.8 (40).

## RESULTS

### Physiological Responses during Experimental Trials

**Figure 2.**
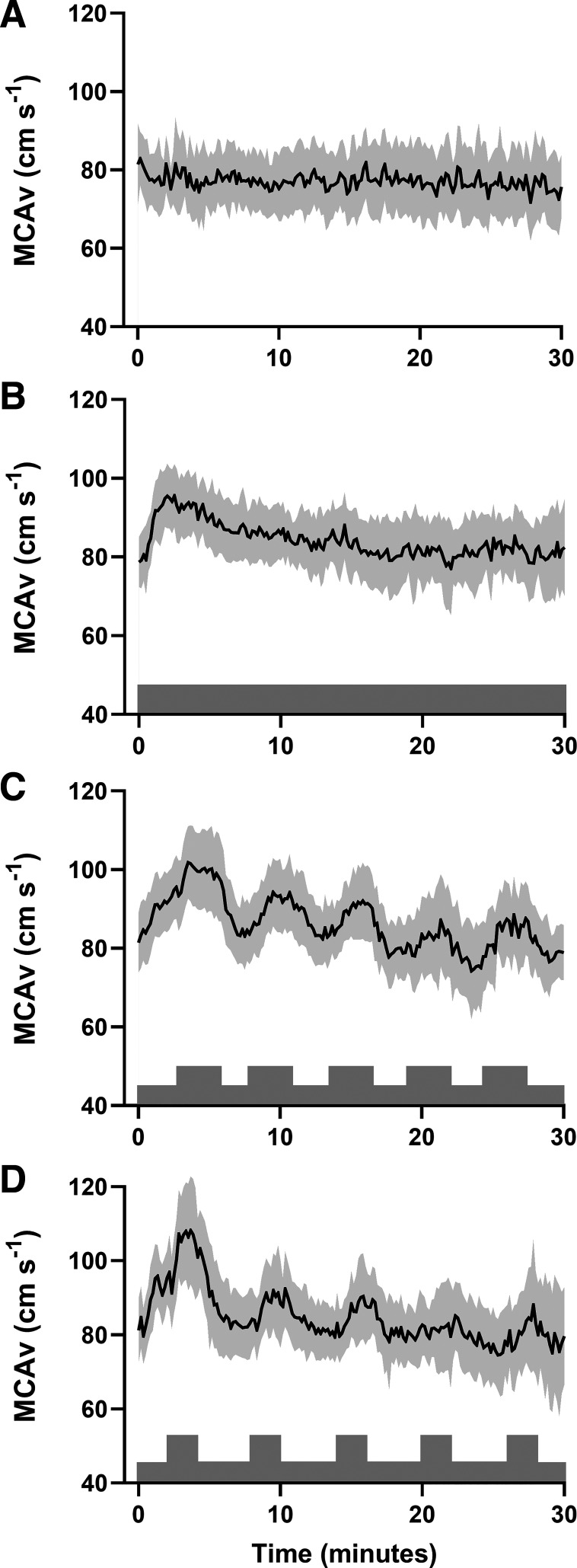
Mean ± 95% CI (shaded) middle cerebral artery blood velocity (MCAv) across the four experimental trials [control (*A*), moderate-intensity exercise (*B*), high-intensity interval exercise 1 (*C*), high-intensity interval exercise 2 (*D*)]. The shading shows the pattern of the exercise stimulus. *n* = 9 (5 female) due to signal loss in 1 participant. Analysis of between trial differences are presented in Table 2. CI, confidence interval.

The group responses to each experimental trial are presented in Table 2 and Fig. 2. The power output for each exercise intensity was as follows: 45% V̇o_2max_ 72 ± 19 W, 60% V̇o_2max_ 118 ± 25 W, 75% V̇o_2max_ 161 ± 29 W, 90% V̇o_2max_ 211 ± 39 W. By design, mean V̇o_2_ was not different between exercise trials (*P* = 0.44, $\eta_{\text{p}}^{2}$ = 0.08), although the highest V̇o_2_ achieved was greatest in HIIE2 compared with HIIE 1 (*P* < 0.001, *d* = 0.58) and MIE (*P* < 0.001, *d* = 1.33), whereas HIIE1 was greater than MIE (*P* < 0.001, *d* = 0.87). Mean $\text{P}\text{E}{\text{T}}_{\text{C}{\text{O}}_{2}}$ was lower in HIIE2 compared with MIE (*P* = 0.007, *d* = 0.75) and HIIE1 (*P* = 0.028, *d* = 0.54). $\text{P}\text{E}{\text{T}}_{\text{C}{\text{O}}_{2}}$ minimum was lower in HIIE2 compared with MIE (*P* < 0.001, *d* = 1.16) and in HIIE1 compared with MIE (*P* = 0.045, *d* = 0.46). Average MCAv mean (*P* = 0.03, *d* = 0.92), MCAv peak (*P* = 0.04, *d* = 0.77), and area under the MCAv mean curve versus time (*P* = 0.02, *d* = 1.11) were greater in HIIE1 compared with CON.

**Table 2.** Physiological responses to the experimental trials

	CON	MIE	HIIE1	HIIE2
V̇o_2_ mean, L/min		1.71 ± 0.35	1.69 ± 0.30	1.68 ± 0.34
V̇o_2_ mean (%V̇o_2max_)		65 ± 4	65 ± 3	64 ± 3
V̇o_2peak_, L/min		1.96 ± 0.35	2.27 ± 0.37^b^	2.52 ± 0.47^b, c^
V̇o_2peak_ (%V̇o_2max_)		75 ± 5	88 ± 8^b^	97 ± 5^b, c^
$\text{P}\text{E}{\text{T}}_{\text{C}{\text{O}}_{2}}$ mean, mmHg		39.4 ± 3.5	38.7 ± 3.6	36.9 ± 2.9^b, c^
$\text{P}\text{E}{\text{T}}_{\text{C}{\text{O}}_{2\text{peak}}}$, mmHg		43.8 ± 4.1	44.7 ± 4.4	44.8 ± 3.9
$\text{P}\text{E}{\text{T}}_{\text{C}{\text{O}}_{2}}$ minimum, mmHg		35.4 ± 3.0	33.7 ± 4.2^b^	31.5 ± 3.6^b^
MCAv mean, cm/s	79.3 ± 12.0	85.5 ± 11.6	89.1 ± 9.1^a^	84.8 ± 13.1
MCAv peak, cm/s	90.9 ± 13.4	105.1 ± 11.3	110.1 ± 10.6^a^	113.4 ± 20.4
MCAv AUC, cm/s × 30 min	2382 ± 413	2516 ± 405	2757 ± 235^a^	2590 ± 441

V̇o_2peak_, $\text{P}\text{E}{\text{T}}_{\text{C}{\text{O}}_{2\text{peak}}}$, and $\text{P}\text{E}{\text{T}}_{\text{C}{\text{O}}_{2}}$ minimum reflect the highest and lowest 10-s averages, respectively. Due to signal loss in 1 participant, *n* = 9 for MCAv outcomes. ^a^Significantly different from CON. ^b^Significantly different from MIE. ^c^Significantly different from HIIE1. AUC, area under the blood flow versus time (30 min) curve; CON, control; HIIE, high-intensity interval exercise; MCAv, blood velocity in the middle cerebral artery; MIE, moderate-intensity exercise; $\text{P}\text{E}{\text{T}}_{\text{C}{\text{O}}_{2}}$, partial pressure of end tidal carbon dioxide; V̇o_2_, oxygen consumption; V̇o_2max_, maximum oxygen uptake from prior incremental test.

### Peripheral Vascular Outcomes

**Figure 3.**
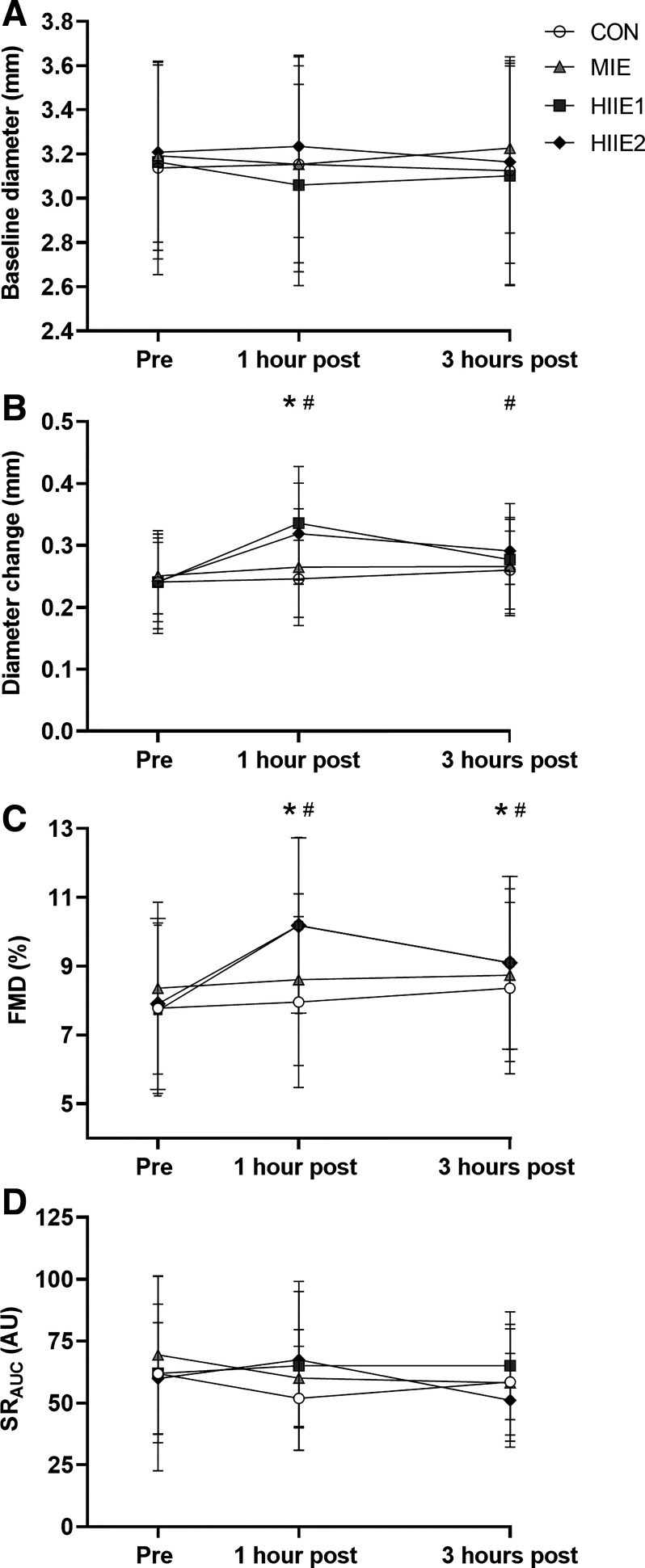
Peripheral vascular outcomes [baseline diameter (*A*), absolute change in brachial artery diameter (*B*), allometrically adjusted FMD (*C*), area under the shear rate curve until time of peak dilation (*D*)] before (pre) and 1 and 3 h after the four experimental trials (*n* = 10, 6 female). A repeated-measures ANOVA revealed a significant time by trial interaction effect for the change in brachial artery diameter after occlusion (*P* = 0.025; *B*) and the allometrically adjusted flow-mediated dilation statistic (*P* = 0.014; *C*). Only the within-trial significant differences are denoted. *Different from pre within the high-intensity interval trial 1. #Significantly different from pre within the high-intensity interval trial 2. CON, control; FMD, flow-mediated dilation allometrically adjusted to baseline diameter; HIIE, high-intensity interval exercise; MIE, moderate-intensity exercise; SR_AUC_, area under the shear rate curve until time of peak dilation.

There was a time by trial interaction effect for brachial baseline diameter (*P* = 0.002, η^2^ = 0.305, Fig. 3*A*). Baseline diameter was never different from pre within a trial; however, baseline diameter was lower 1 h after HIIE1 compared with 1 h after CON (*P* = 0.029, *d* = 0.20) and HIIE2 (*P* = 0.003, *d* = 0.40).

There was a time by trial interaction for the absolute change in brachial artery diameter after occlusion (*P* = 0.025, η^2^ = 0.321 Fig. 3*B*). Within trial, the change in brachial artery diameter was greater 1 h (*P* = 0.001, *d* = 1.09) after HIIE1 compared with pre. In the HIIE2 trial, the change in brachial artery diameter was greater 1 h (*P* < 0.001, *d* = 0.97) and 3 h (*P* = 0.032, *d* = 0.74) after HIIE2 compared with pre. Between trials, there were no differences between trials at the pre time point (*P* > 0.368, *d* < 0.16). One hour after exercise, the change in brachial artery diameter after occlusion was greater in the HIIE1 trial than CON (*P* = 0.002, *d* = 1.15) and MIIE (*P* = 0.006, *d* = 0.76) but not different compared with HIIE2 (*P* = 0.442, *d* = 0.23). The change in brachial diameter after occlusion was only different between HIIE2 and CON at the 3 h time point (*P* = 0.033, *d* = 0.53).

There was no main effect of trial (*P* = 0.996, η^2^ = 0.002), time (*P* = 0.707, η^2^ = 0.038), or trial by time interaction (*P* = 0.382, η^2^ = 0.108) for the time taken to achieve peak dilation after occlusion.

There was a time by trial interaction for allometrically adjusted FMD (*P* = 0.014, Fig. 3*C*). Within trial, FMD was never different from pre values at any time points in the CON (*P* > 0.325) or MIE (*P* > 0.521) trials. In the HIIE1 trial, FMD was elevated 1 h (*P* < 0.001, *d* = 1.13) and 3 h (*P* = 0.023, *d* = 0.54) after HIIE1 compared with pre. In the HIIE2 trial, FMD was elevated 1 h (*P* < 0.001, *d* = 0.91) and 3 h (*P* = 0.043, *d* = 0.48) after HIIE2 compared with pre.

Between trials, there were no differences in FMD between trials at the pre time point (*P* > 0.322, *d* < 0.26). One hour after exercise, FMD in the HIIE1 trial was greater than in CON (*P* < 0.001, *d* = 1.01) and greater than in MIIE (*P* = 0.002, *d* = 0.77) but not different compared with HIIE2 (*P* = 0.544, *d* = 0.14). FMD was also greater 1 h after HIIE2 compared with the same time point in CON (*P* < 0.001, *d* = 0.88) and greater than MIE (*P* = 0.010, *d* = 0.62). No differences between trials were observed at the 3 h time point (*P* > 0.210, *d* < 0.30 for all).

Mean SR_AUC_ data are presented in Fig. 3*D* (*n* = 9 due to signal loss). There was no effect of trial (*P* = 0.466, η^2^ = 0.099), time (*P* = 0.592, η^2^ = 0.046), or trial by time interaction effect (*P* = 0.312, η^2^ = 0.132) for SR_AUC_.

### Cerebrovascular Outcomes

**Figure 4.**
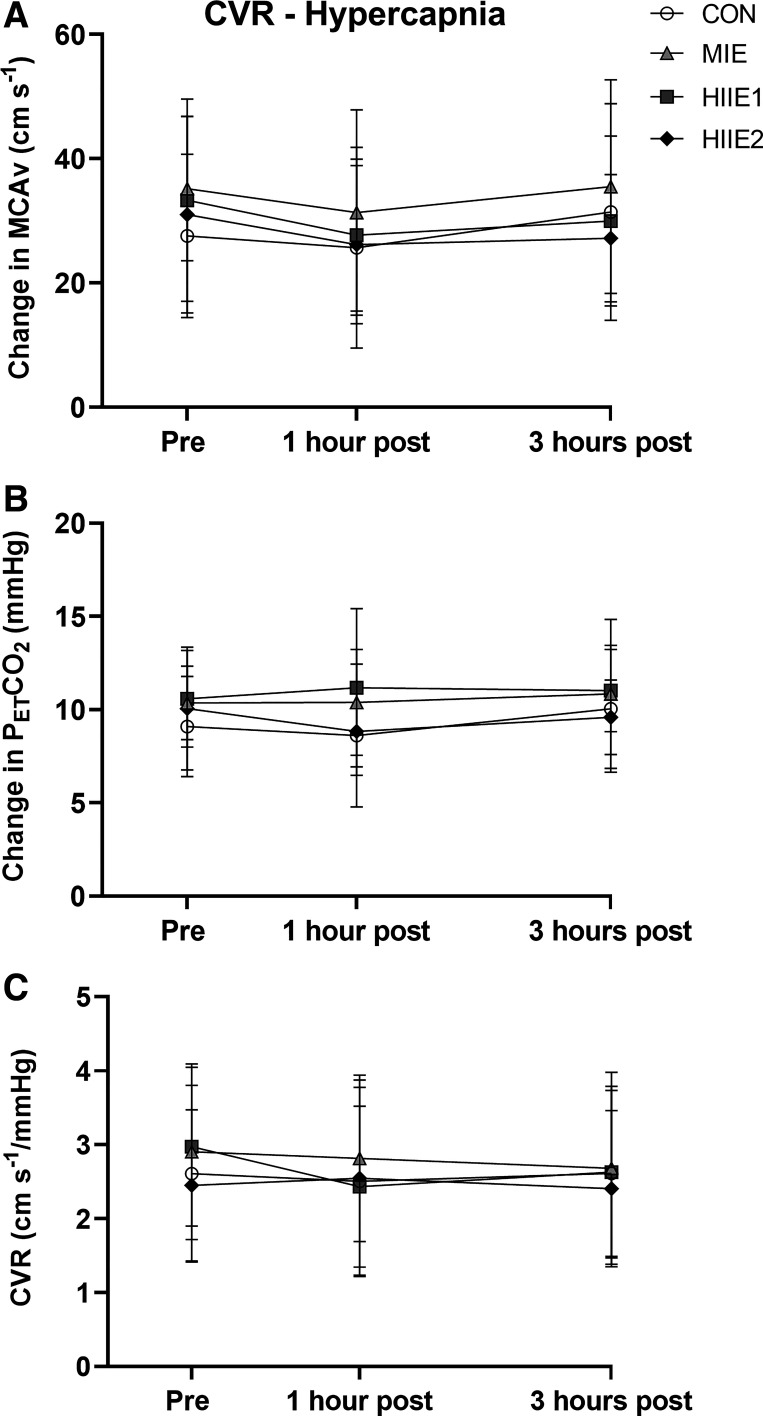
Physiological responses to 5 min of 5% carbon dioxide inhalation (*n* = 10, 6 female). *A*: MCAv change reflects the difference between resting baseline and the highest 30 s rolling average during the hypercapnic stimulus. *B*: the $\text{P}\text{E}{\text{T}}_{\text{C}{\text{O}}_{2}}$ change is calculated as the difference between baseline and this time point. *C*: cerebrovascular reactivity A repeated-measures ANOVA revealed no significant time by trial interaction for any of these outcomes (*P* > 0.605, η^2^ < 0.078). CON, control; CVR, cerebrovascular reactivity; HIIE, high-intensity interval exercise; MCAv, middle cerebral artery blood velocity; MIE, moderate-intensity exercise; $\text{P}\text{E}{\text{T}}_{\text{C}{\text{O}}_{2}}$, end tidal partial pressure of carbon dioxide.

There was no trial by time interaction for resting MCAv (*P* = 0.713, η^2^ = 0.064, Fig. 4*A*); however, there was a main effect of time (*P* = 0.02, η^2^ = 0.379). Resting MCAv was higher at the pre time point compared with 1 h post (*P* = 0.01, *d* = 0.39) and 3 h post (*P* = 0.01, *d* = 0.51) across the experimental trials.

There was no main effect of trial (*P* = 0.789, η^2^ = 0.042), time (*P* = 0.093, η^2^ = 0.257), or trial by time interaction (*P* = 0.379, η^2^ = 0.118) for resting mean arterial pressure. There was no main effect of trial (*P* = 0.712, η^2^ = 0.054), time (*P* = 0.170, η^2^ = 0.217), or trial by time interaction for resting CVC (*P* = 0.207, η^2^ = 0.175).

### Cerebrovascular Reactivity

The responses to the hypercapnic stimulus are presented in Fig. 4. A mean increase in $\text{P}\text{E}{\text{T}}_{\text{C}{\text{O}}_{2}}$ of 10.1 ± 3.0 mmHg was observed at the time of peak MCAv, which was never different across observations (*P* = 0.722, η^2^ = 0.063). There was no time by trial interaction for the increase in MCAv during the hypercapnic challenge (*P* = 0.820, η^2^ = 0.051). There was no main effect of trial (*P* = 0.135, η^2^ = 0.183), time (*P* = 0.145, η^2^ = 0.193), or trial by time interaction (*P* = 0.605, η^2^ = 0.078) for CVR hypercapnia.

**Figure 5.**
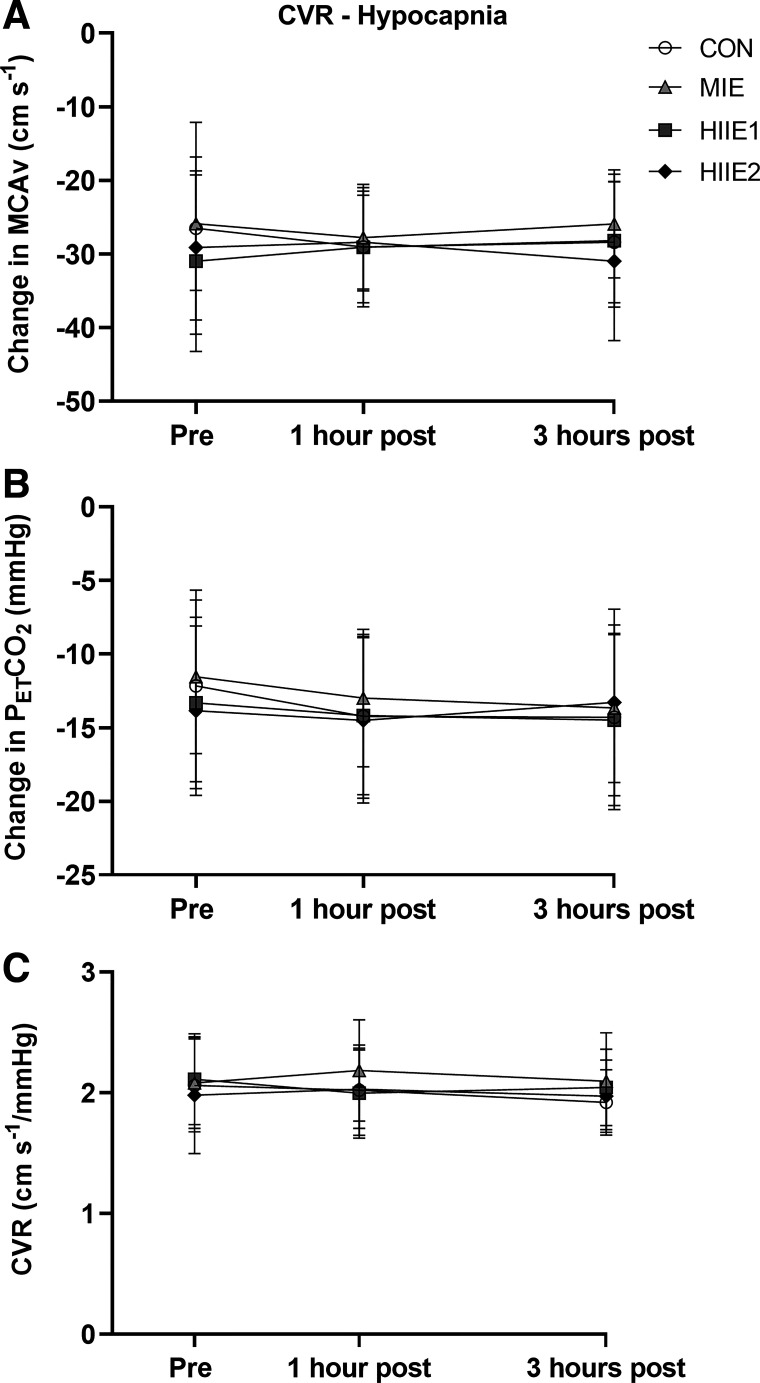
Physiological responses to 1 min of deep hyperventilation (*n* = 10, 6 female). The change in MCAv (*A*) and $\text{P}\text{E}{\text{T}}_{\text{C}{\text{O}}_{2}}$ (*B*) reflects the difference between resting baseline and the final 10 s of the hyperventilation stimulus, used to calculate cerebrovascular reactivity (*C*). A repeated-measures ANOVA revealed no significant time by trial interaction for any of these outcomes (*P* > 0.594, η^2^ < 0.079). CON, control; CVR, cerebrovascular reactivity; HIIE, high-intensity interval exercise; MCAv, middle cerebral artery blood velocity; MIE, moderate-intensity exercise; $\text{P}\text{E}{\text{T}}_{\text{C}{\text{O}}_{2}}$, end tidal partial pressure of carbon dioxide.

Figure 5 presents the physiological responses during the hyperventilation challenge. This stimulus caused a mean reduction in $\text{P}\text{E}{\text{T}}_{\text{C}{\text{O}}_{2}}$ of 13.5 ± 5.5 mmHg, which was never different across observations (time by trial interaction *P* = 0.594, η^2^ = 0.060). There was no time by trial interaction for the fall in MCAv during hyperventilation (*P* = 0.763, η^2^ = 0.053). There was no main effect of trial (*P* = 0.157, η^2^ = 0.173), time (*P* = 0.688, η^2^ = 0.041), or trial by time interaction (*P* = 0.595, η^2^ = 0.079) for CVR hypocapnia.

## DISCUSSION

This study found that HIIE (with exercise intervals performed at both 75% and 90% V̇o_2max_) acutely improved brachial artery FMD in healthy young adults, compared with MIE and a sedentary control condition. However, the present study found no effect of exercise intensity on CVR, with CVR to both hyper- and hypocapnia remaining unchanged following the exercise and sedentary control conditions. Finally, the present study found no significant differences in the overall MCAv response to exercise between exercise conditions (expressed as MCAv AUC), although the profile of MCAv during exercise differed between protocols.

### The Effect of Exercise Intensity on Peripheral Vascular Function

The present study found that allometrically adjusted brachial artery FMD was significantly elevated above baseline 1 and 3 h following both HIIE conditions, but continuous MIE did not alter FMD in healthy adults. These findings are in agreement with previous work in healthy adults comparing high- and moderate-intensity constant work-rate exercise (9). These findings are also consistent with previous data in healthy adolescents, where HIIE performed at 90% peak power significantly elevated brachial artery FMD compared with work-matched MIE 1 h following exercise (27). The present study develops these findings and shows that elevations in FMD extend for 3 h post-HIIE in healthy adults. Exercise-induced elevations in shear stress have been shown to mediate postexercise improvements in brachial artery FMD (3, 23). Given this, the superior improvement in postexercise FMD during the HIIE trials, compared with MIE, is likely a consequence of greater brachial artery shear stress during higher exercise intensities (6, 7), or an important role of the interval pattern of exercise (41). Interestingly, the present study found no further benefit of work-matched HIIE performed at 90% V̇o_2max_, compared with 75% V̇o_2max_, but this may be a consequence of matching the exercise conditions (i.e., greater intensity at 90% V̇o_2max_, but shorter interval duration). It is possible this may have resulted in a similar shear stress stimulus between the two HIIE trials, where the same number of intervals were also performed. However, it was not possible to measure brachial artery shear stress during the exercise conditions, and technical challenges limit the ability to measure limb shear rates at such high intensities. Furthermore, other factors have previously been shown to influence postexercise changes in FMD (8), such as differences in redox state between exercise conditions (22) alongside participant characteristics, such as cardiorespiratory fitness (42). Nevertheless, the present data show acute beneficial effects of HIIE on peripheral vascular function in healthy adults, compared with equivalent continuous moderate-intensity exercise.

### The Effect of Exercise Intensity on Cerebrovascular Reactivity

Contrary to the hypothesis, the present study found that CVR was unaltered following any exercise condition in healthy adults. Given that peripheral endothelial function was increased by HIIE, these data could indicate that the baseline cerebrovascular vasodilatory capacity in healthy young adults cannot be acutely improved by such exercise. This inference is supported by recent evidence that habitual endurance or resistance training in young adults was also not associated with elevated CVR compared with untrained young, healthy adults (aged ∼28 yr) (43). It remains to be explored if acute exercise can have a positive effect on cerebrovascular function in populations with impaired CVR, such as older adults or individuals with cerebrovascular disease. Exercise training has been shown to improve CVR in stroke patients (44), and a recent systematic review found that exercise training tended to improve CVR in older adults, although data are limited to just four studies (45). Collectively, these data suggest that exercise does not acutely or chronically elevate CVR in a sample of healthy young adults, where benefits may be observed in older or at-risk populations.

The data from the present study are in contrast to the only previous study investigating CVR following acute HIIE, where Burma et al. (20) observed a 37% decrease in CVR to hypercapnia immediately and 1 h following exercise (ten 1-min intervals at ∼85% heart rate reserve), also in young adults. This was suggested to be a consequence of repeated and prolonged cerebral vasoconstriction that occurred during HIIE, as a result of hyperventilation-induced hypocapnia (21), subsequently altering vessel tone and limiting the capacity of the cerebrovasculature to maximally dilate. However, the present study suggests that the vasodilatory capacity of the cerebrovasculature is preserved following both HIIE protocols. The differences between the findings from Burma et al. and those of the present study may be attributed to the HIIE protocol used (i.e., a different intensity and number of intervals used) or in the technique used to assess CVR. The present study delivered a steady-state increase in $\text{P}\text{E}{\text{T}}_{\text{C}{\text{O}}_{2}}$ through fixed concentration CO_2_ breathing, whereas Burma et al. used a rebreathing protocol, which elevates $\text{P}\text{E}{\text{T}}_{\text{C}{\text{O}}_{2}}$ breath-by-breath and elicits much larger increases in $\text{P}\text{E}{\text{T}}_{\text{C}{\text{O}}_{2}}$ (∼30 mmHg) compared with the current study (∼10 mmHg). This is an important consideration given the different limitations of different methods used to assess CVR (46). As highlighted by a recent systematic review, the effects of HIIE on cerebrovascular function are poorly understood (16) and are likely influenced by protocol, assessment method, and population.

It was hypothesized that HIIE performed at 75% V̇o_2max_ would elicit the greatest increases in MCAv during exercise, compared with HIIE performed at 90% V̇o_2max_ and MIE at 60% V̇o_2max_, as this is the intensity that is thought to elicit the greatest increases in MCAv during incremental exercise (25). It was also hypothesized that this would result in the greatest postexercise improvements in CVR, as shear stress and elevations in cerebral blood follow during exercise are thought to be important mechanisms for exercise-induced improvements in cerebrovascular reactivity (14, 23). However, only HIIE performed at 75% V̇o_2max_ elicited an MCAv AUC response greater than the seated control condition, and this could be underpinning the absence of change in postexercise CVR observed in the present study. Consequently, the exercise protocols used in the present study may not have provided a sufficient stimulus to mediate postexercise improvements in CVR. However, cerebral blood velocity is not the same as shear stress, and without a measure of shear during exercise, this remains speculative. Some pilot data suggest that exercise-induced elevations in shear stress in the internal carotid artery (ICA) are almost double during HIIE compared with work-matched MIE (14), but these data are very limited and likely protocol-dependent. Furthermore, resting shear stress of the ICA is very high in young adults (over fourfold that of the brachial artery) (18). Therefore, it is also possible that resting characteristics of the cerebrovasculature in this population, particularly the high levels of baseline shear, limit the capacity for exercise to further elevate shear stress and thus improve cerebrovascular reactivity via this mechanism. Nevertheless, future research is needed to first understand the CBF and shear stress responses to different exercise conditions and then to investigate the subsequent effects on cerebrovascular function.

A key observation from the present study was that HIIE improved peripheral vascular function, but that this was not mirrored by changes in cerebrovascular reactivity in healthy young adults. Initial evidence suggested that the overnight changes in the two were related (17), but more recently Carr et al. (18) observed no significant correlation between resting peripheral and cerebral shear-mediated endothelial function at rest. The present study provides further evidence that the peripheral and cerebral vascular systems may share a different mechanism of change, building on previous data from our laboratory, where sugar sweetened-beverage consumption increased brachial artery FMD but did not change CVR in adolescents (29). Collectively, these data provide further support that findings from the periphery cannot be extrapolated to cerebrovascular reactivity. Nevertheless, the present study provides valuable and novel data that further contribute to a growing discussion around HIIE and cerebrovascular health (14–16).

### Study Considerations

The present study has a number of methodological strengths. These include the time- and average intensity-matching of the three exercise conditions and the inclusion of a control condition, allowing thorough investigation into the effect of exercise intensity. Furthermore, all data were analyzed according to published guidelines, with FMD data scaled allometrically (47) and CVR analyzed using a reliable approach from open circuit CO_2_ breathing tests (37).

However, the present study is not without its limitations. This study had six female participants, and menstrual cycle phase was not controlled for in this study. Whether the menstrual cycle should be controlled for in cardiovascular research has been debated (48, 49). Although there is some evidence to suggest that cardiovascular outcomes are influenced by menstrual cycle phase (49), recent research has found no effect of menstrual cycle phase on allometrically scaled brachial artery FMD (50) nor cerebral autoregulation in healthy, young females (51, 52). Nevertheless, whether the menstrual cycle alters the acute FMD response to exercise is unknown.

This study used TCD to measure MCAv, which is only a valid surrogate measure of cerebral blood flow if MCA diameter remains unchanged (53). Although debated (54, 55), TCD is considered an appropriate and practical measurement technique to assess CBF during exercise and CO_2_ breathing challenges (53), though there is a lack of standardization for TCD-measured CVR regarding protocol and data handling, which introduces conflict in the existing literature (39). Furthermore, assessing CVR in this way introduces greater variability, compared with targeting $\text{P}\text{E}{\text{T}}_{\text{C}{\text{O}}_{2}}$ levels using end-tidal forcing (56). Future studies using end-tidal forcing and MR techniques are therefore warranted. Nevertheless, open-circuit CO_2_ breathing remains a commonly used method to assess CVR, and data have been analyzed using the most reliable approach to minimize the potentially confounding effects of differences in ventilation and $\text{P}\text{E}{\text{T}}_{\text{C}{\text{O}}_{2}}$ within and between day (37).

An additional consideration is the timing of postexercise assessments of vascular function, with measurements completed at 1 and 3 h after exercise. This means the time-course of potential postexercise changes in CVR cannot be fully determined, which is an important consideration given that Burma et al. (20) observed a significant decrease in CVR immediately following HIIE. However, we can confirm that any postexercise changes in CVR do not coincide with alterations in FMD, so the possibility that changes in CVR were missed in our study is unlikely.

An additional limitation is the inclusion of intracranial measures of the MCA only. Marked regional differences have been observed in the CBF response to incremental exercise, with ICA and MCA blood flow decreasing after ∼70%–75% V̇o_2max_, whereas blood flow in the vertebral and posterior cerebral artery continues to increase with greater exercise intensity (25, 57). It has therefore been suggested that HIIE may elicit greater positive effects on the posterior circulation (14), which is more susceptible to deterioration than the anterior circulation (58). However, Labrecque et al. (59) observed similar responses of the middle and posterior cerebral arteries during and following a single bout of HIIE, performed at 100% V̇o_2max_ in young, fit women. Furthermore, Burma et al. (20) found similar post-HIIE CVR responses in the middle and posterior cerebral arteries. Collectively, existing data suggest more research is needed on the regional cerebrovascular responses during and following acute HIIE (16). Finally, CVR measured in the MCA is not the same as ICA shear-mediated function (18), which may show differential responses following HIIE, particularly if shear stress is a primary mechanism and therefore requires further investigation.

### Conclusions

This study found that acute HIIE performed with intervals at 75% and 90% V̇o_2max_ improved peripheral vascular function 1 and 3 h following exercise in healthy adults, which was unaltered by MIE. However, CVR of the MCA was not altered following either HIIE conditions or MIE. These novel data show distinctly different responses of the peripheral and cerebral vasculature following HIIE and provide valuable information on the effect of HIIE on cerebrovascular reactivity. These data contribute to a growing discussion surrounding high-intensity exercise and the brain (14, 15), with future research needed to explore potentially important considerations surrounding the exercise dose (intensity, duration), measurement technique and timing, and sample used.

## GRANTS

This work was funded by the Physiological Society (Grant Number: 29389-FR).

## DISCLOSURES

No conflicts of interest, financial or otherwise, are declared by the authors.

## AUTHOR CONTRIBUTIONS

A.R.B. and B.B. conceived and designed research; M.E.W., J.L.K., and A.B.L. performed experiments; M.E.W., J.L.K., A.B.L., and B.B. analyzed data; M.E.W., J.L.K., A.B.L., A.R.B., and B.B. interpreted results of experiments; M.E.W. and B.B. prepared figures; M.E.W. drafted manuscript; M.E.W., J.L.K., A.B.L., A.R.B., and B.B. edited and revised manuscript; M.E.W., J.L.K., A.B.L., A.R.B., and B.B. approved final version of manuscript.
